# Dancing to wellbeing: a three-wave longitudinal mixed-methods study of Rock “n” Roll Dancesport and psychosocial outcomes in Chinese university students

**DOI:** 10.3389/fpubh.2026.1928623

**Published:** 2026-10-02

**Authors:** Yuanmei He, Jialin Wu

**Affiliations:** School of Physical Education, Guangzhou University, Guangzhou, China

**Keywords:** anxiety symptoms, Chinese university students, heart rate variability, longitudinal mixed-methods, Rock “n” Roll Dancesport, social self-efficacy

## Abstract

**Background:**

Mental health challenges among university students have intensified globally, with rising anxiety and social isolation reported across diverse educational contexts. These trends demand innovative, accessible, and non-stigmatizing approaches that complement traditional clinical services. This study examined whether participation in Rock “n” Roll Dancesport (Jive and Cha-Cha) was prospectively associated with improvements in self-efficacy, anxiety symptoms, and heart rate variability (HRV) among Chinese university students over 6 months.

**Methods:**

A three-wave longitudinal mixed-methods design was employed. Quantitative assessments were conducted at baseline (T1, *n* = 152), 3 months (T2, *n* = 136), and 6 months (T3, *n* = 131). Measures included the General Self-Efficacy Scale (GSES), Social Self-Efficacy Scale (SSES), GAD-7 for anxiety symptoms, and resting HRV. Linear mixed-effects models examined the effects of time, baseline dance frequency group (high: ≥3 sessions/week vs. low: 1–2 sessions/week), and their interaction on each outcome, controlling for age, gender, and academic year. Semi-structured interviews (*n* = 32) and 50 + hours of observation explored participants’ lived experiences.

**Results:**

Mixed-effects models revealed significant main effects of frequency group across all outcomes: high-frequency participants showed higher GSES (*β* = 3.60, *p* < 0.001), higher SSES (*β* = 8.62, *p* < 0.001), lower GAD-7 (*β* = −2.80, *p* < 0.001), and higher HRV (*β* = 7.64 ms, *p* < 0.001). Significant Time × Frequency Group interactions indicated that high-frequency participants showed greater improvements over 6 months in SSES (T2: *β* = 2.40, *p* = 0.001; T3: *β* = 2.37, *p* = 0.001), GSES (T3: *β* = 0.99, *p* = 0.007), and HRV (T3: *β* = 3.10, *p* = 0.004). Qualitative analysis identified four interconnected experiential themes: rhythmic entrainment as affective catalyst, embodied mastery as a source of self-efficacy, synchronized partnership as a context for trust, and meaning-making as narrative integration.

**Conclusion:**

More frequent Rock “n” Roll Dancesport participation was prospectively associated with improvements in self-efficacy, anxiety symptoms, and HRV over 6 months. The convergence of quantitative trajectories and qualitative narratives supports a directional interpretation consistent with participation temporally preceding psychological gains, while acknowledging that self-selection and reciprocal effects remain plausible. These findings suggest that structured, arts-based physical activities may serve as promising, non-stigmatizing contexts for resource-building among university students.

## 1 Introduction

The mental health of university students has become a growing concern in higher education worldwide. Systematic reviews and multinational surveys indicate that between 20 and 35% of university students report clinically significant levels of anxiety, depression, or related psychological distress across diverse cultural contexts (1). This burden is multifactorial, stemming from academic pressure, social isolation, financial strain, and developmental challenges associated with young adulthood. These trends are compounded by a global decline in physical activity among young adults and increased engagement with sedentary, screen-based behaviors (2).

Within the Chinese higher education system, these challenges are particularly pronounced. Recent systematic reviews have documented sustained high prevalence of depression, anxiety, and sleep disturbances among university students in the post-pandemic period (3). These difficulties are intensified by academic competition—often described as “involution”—and limited opportunities for meaningful social connection on large, impersonal campuses. In this context, wellbeing activities that are accessible, culturally situated, and aligned with students’ existing interests may offer a complementary pathway to formal mental health services, potentially reducing barriers related to stigma and access.

This study examined a specific activity: regular participation in Dancesport—Jive and Cha-Cha—performed to Rock “n” Roll music. We conceptualized this practice as a structured, culturally situated activity that integrates psychological, social, and physiological processes relevant to wellbeing. Unlike individual forms of exercise, Dancesport requires rhythmic coordination, embodied skill acquisition, and sustained interpersonal cooperation, all of which may contribute to psychosocial adaptation through distinct yet interacting mechanisms.

Systematic reviews and meta-analyses have demonstrated that dance interventions are effective in improving psychological outcomes across diverse populations (4). Koch and colleagues (4) found that dance interventions positively affect aspects of participants’ self, including self-concept, self-esteem, and body image. Meta-analytic evidence further indicates that structured dance is at least as effective as other forms of physical activity in improving psychological and cognitive health outcomes (5), and in some comparisons—particularly for reducing anxiety and depression—dance interventions show superior effects (6). Dance movement therapy and dance have been shown to significantly improve health-related psychological outcomes, with moderate to large effect sizes for quality of life and well-being (7, 8).

Our investigation was guided by two complementary theoretical frameworks. The first is Fredrickson’s broaden-and-build theory of positive emotions (9, 10), which proposes that positive affective states broaden momentary thought-action repertoires and, over time, build enduring personal resources across psychological, social, and physical domains. The second is the personal resource mediation model demonstrated by Wang, Lü, Du, and Wang among Chinese undergraduates (11), which found that the effect of positive emotions on mental health was fully mediated by personal resources including psychological resilience, positive coping, interpersonal trust, and perceived social support.

While previous research has established the general architecture of this pathway, less is known about how these mechanisms unfold over time in naturalistic settings. Laboratory experiments and cross-sectional surveys have provided evidence for the statistical mediation model, but they offer limited insight into how resources are built, embodied, and sustained through sustained engagement in daily life. This study addressed this gap by following participants over 6 months, examining both the longitudinal trajectories of psychosocial outcomes and the experiential processes participants identified as relevant to their experiences.

This study addressed two research questions:

(RQ1) Is the frequency of Rock “n” Roll Dancesport participation prospectively associated with differential change in self-efficacy, anxiety symptoms, and resting HRV over 6 months?

(RQ2) What experiential processes do participants identify as underlying their perceived changes over time?

## 2 Theoretical framework

### 2.1 Empirical foundation

The resource-mediation model demonstrated by Wang et al. (11) provided the empirical foundation for this study. Their analysis of 877 Chinese undergraduates showed that a higher-order latent factor comprising resilience, adaptive coping, interpersonal trust, and perceived social support fully mediated the relationship between positive emotions and mental health outcomes. This finding established that the pathway from positive affective experience to psychological adaptation operates through the accumulation of personal resources, and that these resources—rather than positive emotions alone—are the proximal determinants of wellbeing.

This model raises a practical question: if personal resources mediate the pathway to wellbeing, what kinds of activities are effective at building those resources? The present study examined whether a structured, socially embedded physical practice could serve this function.

### 2.2 A theoretical synthesis

We integrated three theoretical perspectives to explain how Rock “n” Roll Dancesport might build personal resources over time. This integration is proposed as a novel synthesis, as no existing study has directly combined these frameworks in a longitudinal context.

Rhythmic Entrainment. Thaut, McIntosh, and Hoemberg (12) demonstrated that external auditory rhythms can entrain internal biological oscillators, influencing motor timing, autonomic function, and affective states. The function of rhythmic entrainment in rehabilitative training and learning was established in the early 1990s. Temporal rhythmic entrainment has been successfully extended into applications in cognitive rehabilitation and speech and language rehabilitation, becoming one of the major neurological mechanisms linking music and rhythm to brain rehabilitation (12). The driving rhythm of Rock “n” Roll music may interrupt cognitive rumination, shift attention to external stimuli, and induce positive affective arousal. In the broaden-and-build framework, this affective shift constitutes the initial “broadening” that enables subsequent resource-building. Dalla Bella and colleagues (13) further elucidated the multi-scale mechanisms underlying beat perception and movement, from physical principles to brain dynamics, providing a comprehensive framework for understanding how auditory rhythms entrain motor behavior.

Embodied Cognition. Shapiro (14) and Fuchs and Koch (15) argued that cognitive and emotional processes are grounded in bodily states and sensorimotor experience. There is a growing body of research indicating that bodily sensation and behavior strongly influence emotional reactions toward situations or objects (15). Motion and emotion are intrinsically connected: one is moved by movement and moved to move (16). Learning the coordinated movements of Jive and Cha-Cha is not merely motor skill acquisition; it is a form of somatic learning that shapes self-concept, emotional awareness, and perceived capability. Confidence gained from progressive body mastery may generalize to other domains. Basso and colleagues (16, 17) proposed the “Synchronicity Hypothesis of Dance,” which posits that humans dance to enhance intra and inter-brain synchrony, involving seven neurobehavioral domains: sensory, motor, cognitive, social, emotional, rhythmic, and creative.

Social Synchrony. Tarr, Launay, and Dunbar (18) showed that coordinated, synchronous movement with others enhances social bonding, cooperation, trust, and perceived closeness. Interpersonal synchrony leads to “self-other” merging, and endorphins are released during executive rhythmic activities including musical interaction (18). In a subsequent experimental study, Tarr, Launay, Cohen, and Dunbar (19) demonstrated that dancing in synchrony leads to elevated pain thresholds and social closeness, providing direct evidence for endorphin release as a mechanism underlying dance-induced social bonding. Cross, Turgeon, and Atherton (20) further demonstrated that interpersonal entrainment has a wide variety of social consequences that span far beyond those that could be considered purely pro-social. A meta-analysis by Mogan, Fischer, and Bulbulia (21) confirmed that synchrony exerts significant effects on prosocial behavior, perceived social bonding, social cognition, and positive affect, providing robust quantitative evidence for the social consequences of interpersonal coordination. Bigand and colleagues (22) recently revealed the geometric organization of interpersonal synchrony in human dance, finding that both music and partners drive synchrony through distinct dance moves—anteroposterior movements such as head bobs synchronize through music, while hand gestures and full-body lateral movements synchronize through visual contact. The neurobiological underpinnings of dance-induced social bonding include the release of endorphins and the activation of brain regions involved in reward and social cognition (16). Partner dance requires constant non-verbal negotiation and shared goal pursuit, generating a sense of shared agency and interpersonal trust.

The integration of these three lenses suggests a sequential process: rhythmic entrainment generates positive affect that enables engagement; embodied mastery builds intrapersonal confidence; synchronized partnership builds interpersonal trust. Over time, repeated engagement in this cycle may lead to the accumulation of durable personal resources that support psychosocial adaptation.

Figure 1 visualizes this theoretical framework.

**Figure 1:**
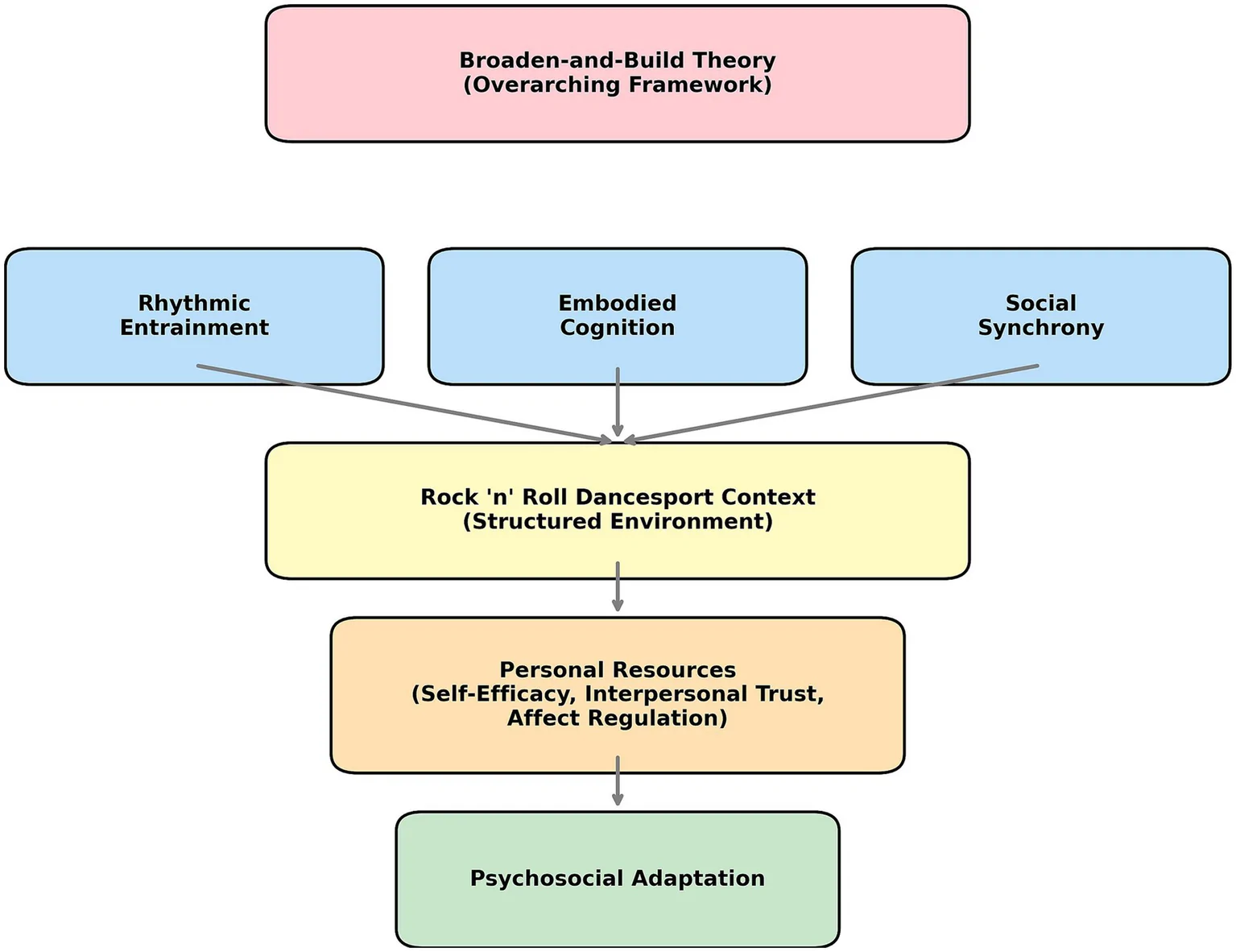
Conceptual framework. The figure illustrates the integrated theoretical model guiding this study. Three theoretical perspectives—rhythmic entrainment (12, 13), embodied cognition (14, 15), and social synchrony (18–22)—converge through the structured context of Rock “n” Roll Dancesport. These processes are hypothesized to generate personal resources (self-efficacy, interpersonal trust, affect regulation), which in turn support psychosocial adaptation. The model situates the broaden-and-build theory (9, 10) as the overarching framework linking momentary affective experiences to durable resource accumulation.

## 3 Methods

### 3.1 Study design and philosophical orientation

This study employed a three-wave longitudinal mixed-methods design with an explanatory sequential structure (QUAN → qual) (23). Quantitative assessments were conducted at baseline (T1), 3 months (T2), and 6 months (T3). The qualitative phase, conducted after T3, explored participants’ lived experiences and perceived changes. The research was guided by a critical realist ontology (24), which acknowledges an objective reality while recognizing that subjective experience is interpretively mediated.

### 3.2 Participants and recruitment

Participants were recruited from four private Dancesport clubs and two university dance societies in a major metropolitan area in Southern China. Inclusion criteria were: (a) current active participation in competitive-style Jive or Cha-Cha with Rock ‘n’ Roll music, and (b) at least one structured practice session per week. Of 187 individuals who expressed initial interest, 152 met eligibility criteria and provided written informed consent at baseline.

Of the 152 baseline participants, 136 completed the T2 assessment (retention: 89.5%), and 131 completed the T3 assessment (retention: 86.2%). A total of 120 participants (78.9%) completed all three waves and formed the core longitudinal analytic sample. Attrition analysis comparing completers (*n* = 120) with non-completers (*n* = 32) showed no significant differences on baseline age, gender, GSES, SSES, GAD-7, HRV, or baseline dance frequency group (all *p* > 0.05), indicating that missingness was not systematically related to study variables. A total of 35 participants were excluded at baseline: 12 did not meet the minimum participation threshold, 8 had incomplete questionnaires, 7 had unusable HRV data, 5 were not practicing with Rock ‘n’ Roll music, and 3 withdrew consent.

At baseline, we further examined the distribution of dance experience among participants. Based on self-report, 56 participants (36.8%) reported having no prior training in Jive or Cha-Cha (i.e., complete beginners); 63 participants (41.4%) reported less than one year of experience; and 33 participants (21.7%) reported one or more years of experience (mean dance tenure: 18.6 ± 8.5 months, as reported in Table 1). In the cross-tabulation of experience level by frequency group, beginners comprised 31.0% (26/84) of the High-frequency group and 44.1% (30/68) of the Low-frequency group; this difference was not statistically significant (*χ*^2^ = 2.73, *p* = 0.10).

**Table 1:** Baseline demographic characteristics and dance practice profile (*N* = 152).

Variable	Category/statistic	*n*/value	Percentage/mean (SD)
Demographics			
Age (years)	Mean (SD)	—	20.58 (± 1.61)
	Range	—	18–24
Gender	Female	88	57.9%
	Male	64	42.1%
Academic year	Freshman	35	23.0%
	Sophomore	52	34.2%
	Junior	41	27.0%
	Senior	24	15.8%
Dance practice			
Dance tenure (months)	Mean (SD)	—	18.6 (± 8.5)
Experience level	Complete beginner	56	36.8%
	< 1 year	63	41.4%
	≥ 1 year	33	21.7%
Baseline Freq_group	High (≥3/week)	84	55.3%
	Low (1-2/week)	68	44.7%
Primary setting	University dance society	68	44.7%
	Private dance club	84	55.3%
Preferred style	Jive	94	61.8%
	Cha-Cha	58	38.2%

SD, Standard deviation. Frequency refers to the number of structured practice sessions per week.

The qualitative subsample (*n* = 32) was purposively selected from the longitudinal sample to ensure maximum variation in gender, age, dance tenure, primary role (lead vs. follow), and trajectory of change.

### 3.3 Measures

#### 3.3.1 Psychometric surveys

General self-efficacy was assessed with the 10-item General Self-Efficacy Scale (GSES) (25). Responses are rated on a 4-point Likert scale from 1 (not at all true) to 4 (exactly true); total scores range from 10 to 40, with higher scores indicating greater general self-efficacy. Social self-efficacy was measured with the 12-item Social Self-Efficacy Scale (SSES) (26), using a 5-point scale from 1 (no confidence) to 5 (complete confidence); total scores range from 12 to 60, with higher scores reflecting greater social confidence. Anxiety symptoms were assessed with the 7-item Generalized Anxiety Disorder scale (GAD-7) (27), with responses from 0 (not at all) to 3 (nearly every day); total scores range from 0 to 21, with higher scores indicating more severe anxiety symptoms. Cronbach’s α values for the present sample were 0.86 (GSES), 0.91 (SSES), and 0.89 (GAD-7).

#### 3.3.2 Dance frequency

Dance frequency was assessed at each wave by the item: “During the past month, how many structured practice sessions did you attend per week on average?” Responses were collected on an ordinal scale (1, 2, 3, 4, or 5 or more sessions per week). This measure did not include unsupervised individual practice. Frequency data were based on self-report and were not independently verified through attendance records. For analysis, participants were grouped into high frequency (≥3 sessions/week) and low frequency (1–2 sessions/week) based on baseline responses.

#### 3.3.3 Heart rate variability

HRV was recorded at each wave between 2:00 p.m. and 4:00 p.m. in a quiet, temperature-controlled room. Participants were asked to refrain from caffeine, alcohol, and vigorous exercise for at least 2 hours prior to recording. A 5-min resting electrocardiogram was obtained using Polar H10 chest straps with participants seated upright and breathing normally. Data were analyzed using Kubios HRV Standard software (version 3.5). RR intervals were visually inspected and automatically corrected using Kubios’ artifact correction algorithm. Recordings with >5% uncorrectable artifacts were excluded. The primary HRV metric was RMSSD (root mean square of successive differences), calculated from raw RR intervals without transformation. No participants reported current use of cardioactive medications or diagnosed cardiovascular conditions.

#### 3.3.4 Propensity score matching and inverse probability weighting

To address potential selection bias arising from systematic baseline differences between frequency groups, we conducted propensity score analyses (28). Propensity scores for being in the High-frequency group (≥3 sessions/week) were estimated via logistic regression using all baseline covariates: age, gender, academic year, dance tenure, and T1 scores on GSES, SSES, GAD-7, and HRV. Two complementary approaches were employed. First, 1:1 nearest-neighbor matching without replacement was performed using a caliper of 0.2 times the standard deviation of the logit-transformed propensity score to create a matched subsample with balanced covariates. Second, inverse probability weighting (IPW) was applied to the full sample, using weights derived from the propensity scores, to estimate the average treatment effect on the treated. Covariate balance was assessed using standardized mean differences (SMD), with SMD < 0.25 considered acceptable (28). In the matched sample, linear mixed-effects models were re-fitted to examine whether the association between frequency group and SSES improvement (T3–T1) remained significant after accounting for observed confounders.

### 3.4 Qualitative data collection

Following the T3 assessment, 32 participants engaged in one-on-one, semi-structured interviews averaging 55 min in duration. The interview guide explored motivations for participation, sensory experiences of rhythm, skill acquisition narratives, partnership dynamics, and perceived changes over the six-month period. Researchers also conducted over 50 h of non-participant observation across the three waves in natural practice settings.

All Dancesport sessions used standard Rock “*n*” Roll music for Jive and Cha-Cha training. Music selection followed World DanceSport Federation (WDSF) competition standards, with Jive music at approximately 176–188 beats per minute (BPM) and Cha-Cha music at approximately 128–136 BPM. Across all observations, instructors used a consistent playlist of classic and contemporary Rock “n” Roll repertoire, including but not limited to “Rock Around the Clock” (Bill Haley), “Johnny B. Goode” (Chuck Berry), “Hound Dog” (Elvis Presley), and contemporary tracks by Taylor Swift (“State of Grace,” “Holy Ground,” “Red,” “Better Than Revenge,” and “Shake It Off”), alongside contemporary Rock “n” Roll-style dance tracks. In practice, tempo and song selection are adapted to students’ learning progress and specific training goals. What was consistently observed across sessions was that the energetic, upbeat character of the music served as an affective catalyst, with participants visibly responding to its rhythmic drive and dynamic contrasts—a “collision” of musical energy and personal enthusiasm that participants themselves described as central to their engagement.

Interviews and observations were conducted by the first author, who had prior qualitative research experience but no formal involvement in the Dancesport community prior to the study. To incorporate reflexivity, the researcher maintained a field journal throughout data collection and analysis. All interviews were conducted in Mandarin Chinese, audio-recorded, and transcribed verbatim. Quotations were translated into English using a forward-backward procedure prioritizing semantic accuracy.

As one participant noted, “just listening to this music gets your blood pumping, but you also have to move your body; listening alone is not enough”—a sentiment that underscores the integral role of embodied movement in translating musical energy into affective experience. The combination of music, physical activity, and group atmosphere, rather than any single element alone, was perceived by participants as central to the wellbeing effects they experienced.

### 3.5 Data analysis

Quantitative Analysis. Data from the longitudinal sample were analyzed using R (version 4.2) and SPSS (Version 28.0). Linear mixed-effects models with random intercepts for participants were fitted using the MIXED procedure in SPSS (version 28.0); R (with the ggplot2 package) was used for data visualization and figure generation. Fixed effects included wave (T1, T2, and T3), baseline frequency group (high vs. low), their interaction, and covariates (age, gender, academic year). The Time × Frequency Group interaction was the primary test of whether baseline participation frequency showed a prospective association with differential change over time. Effect sizes are reported as unstandardized beta coefficients with standard errors. Model assumptions were checked using residual plots and normality tests. All variables showed acceptable skewness and kurtosis values across all three waves (Supplementary Table S3). Given the exploratory nature of the study, no adjustment for multiple comparisons was applied. Sensitivity analyses comparing the core sample (*n* = 120) with the full available sample (*n* = 152) produced consistent results.

Qualitative Analysis. Interview transcripts and field notes were analyzed using Reflexive Thematic Analysis (29, 30). The analytic process involved familiarization, initial coding, theme development, refinement, and narrative synthesis. Preliminary thematic summaries were shared with five participants for feedback. The first author also discussed the emerging theme structure with two colleagues not involved in data collection, which informed refinements to the thematic organization.

### 3.6 Ethical considerations

This study was conducted in accordance with the Declaration of Helsinki. The study protocol was reviewed and approved by the Institutional Review Board of Guangzhou University (Approval No. 2024–023). Written informed consent was obtained from all individual participants included in the study. Participants were fully informed of the study’s purpose, confidentiality procedures, and their right to withdraw at any time.

## 4 Results

### 4.1 Participant characteristics and attrition

Table 1 presents the demographic characteristics and dance practice profile of the baseline sample (*N* = 152). The sample had a mean age of 20.58 years (SD = 1.61), with 57.9% female participants. At baseline, 55.3% were classified as high-frequency participants (≥3 sessions/week) and 44.7% as low-frequency participants (1–2 sessions/week). The majority were recruited from private dance clubs (55.3%) and preferred Jive (61.8%) over Cha-Cha (38.2%). At baseline, dance experience distribution was as follows: 56 participants (36.8%) were complete beginners, 63 (41.4%) had less than one year of experience, and 33 (21.7%) had one or more years of experience. Baseline comparisons between high and low-frequency groups revealed significant differences across all four outcomes, with the high-frequency group consistently reporting more favorable scores (Supplementary Table S1). Baseline correlations among key variables were also examined, with all measures showing moderate intercorrelations in the expected directions (Supplementary Table S2).

Table 2 presents the attrition analysis comparing participants who completed all three waves (*n* = 120) with those who did not (*n* = 32). No significant differences were found between the two groups on any baseline variable (all *p* > 0.05), indicating that attrition was not systematically related to the study variables.

**Table 2:** Attrition analysis: comparison of baseline characteristics between completers and non-completers.

Variable	Completers (*n* = 120)	Non-completers (*n* = 32)	*p*
Age (years)	20.53 (1.63)	20.75 (1.50)	0.50
Gender (% female)	60.0%	50.0%	0.41
T1 GSES	29.06 (3.85)	28.83 (3.20)	0.76
T1 SSES	37.89 (8.20)	39.36 (7.86)	0.36
T1 GAD-7	7.53 (3.49)	7.27 (3.04)	0.70
T1 HRV (ms)	45.90 (11.75)	47.12 (9.78)	0.59
T1 Freq_group (% High)	56.7%	50.0%	0.64

Values are presented as mean (SD) or percentage. *p*-values based on independent-samples *t*-tests (continuous variables) or chi-square tests (categorical variables). All *p* > 0.05, indicating no systematic attrition bias.

### 4.2 Descriptive trends

Table 3 presents the descriptive statistics for all outcomes across the three waves. Over the six-month period, GSES increased from 29.01 to 30.07, SSES increased from 38.20 to 41.94, HRV increased from 46.16 ms to 49.71 ms, and GAD-7 scores decreased from 7.48 to 6.38. These patterns suggested progressive improvements across all four outcomes.

**Table 3:** Descriptive statistics across waves.

Variable	T1 (*n* = 152)	T2 (*n* = 136)	T3 (*n* = 131)
GSES	29.01 ± 3.72	29.15 ± 3.73	30.07 ± 3.87
SSES	38.20 ± 8.12	40.63 ± 8.88	41.94 ± 9.55
GAD-7	7.48 ± 3.39	6.94 ± 3.24	6.38 ± 3.44
HRV (ms)	46.16 ± 11.35	47.99 ± 11.88	49.71 ± 11.76

Values are presented as Mean ± SD. T1 = baseline; T2 = 3-month follow-up; T3 = 6-month follow-up. GSES = General self-efficacy scale; SSES = Social self-efficacy scale; GAD-7 = Generalized anxiety disorder 7-item scale; HRV = Heart rate variability (RMSSD).

### 4.3 Longitudinal trajectories by frequency group

Table 4 displays the three-wave trajectories separately for high-frequency and low-frequency groups. Figure 2 visualizes these trajectories for SSES and GAD-7, illustrating the diverging patterns between groups over time.

**Table 4:** Longitudinal trajectories by Freq_group (M ± SD).

Group	Wave	GSES	SSES	GAD-7	HRV (ms)
High (≥3/wk)	T1	30.6 (3.2)	42.1 (6.2)	6.2 (2.8)	49.4 (11.3)
(*n* = 84)	T2	30.9 (3.1)	46.6 (6.0)	5.7 (2.7)	52.5 (11.7)
	T3	32.0 (2.8)	46.7 (7.0)	5.1 (2.8)	54.3 (10.5)
	Change (T3–T1)	(+1.4)	(+4.6)	*−1.1*	(+4.9)
Low (1–2/wk)	T1	27.0 (3.3)	33.4 (7.6)	9.1 (3.4)	42.1 (10.0)
(*n* = 68)	T2	27.4 (3.6)	34.7 (7.2)	8.2 (3.2)	43.5 (10.4)
	T3	27.2 (3.4)	34.7 (8.3)	8.3 (3.5)	42.8 (10.1)
	Change (T3–T1)	(+0.2)	(+1.3)	*−0.8*	(+0.7)

High = ≥3 structured sessions per week; Low = 1–2 sessions per week. T1 = baseline; T2 = 3 months; T3 = 6 months. Values are mean (SD).

**Figure 2:**
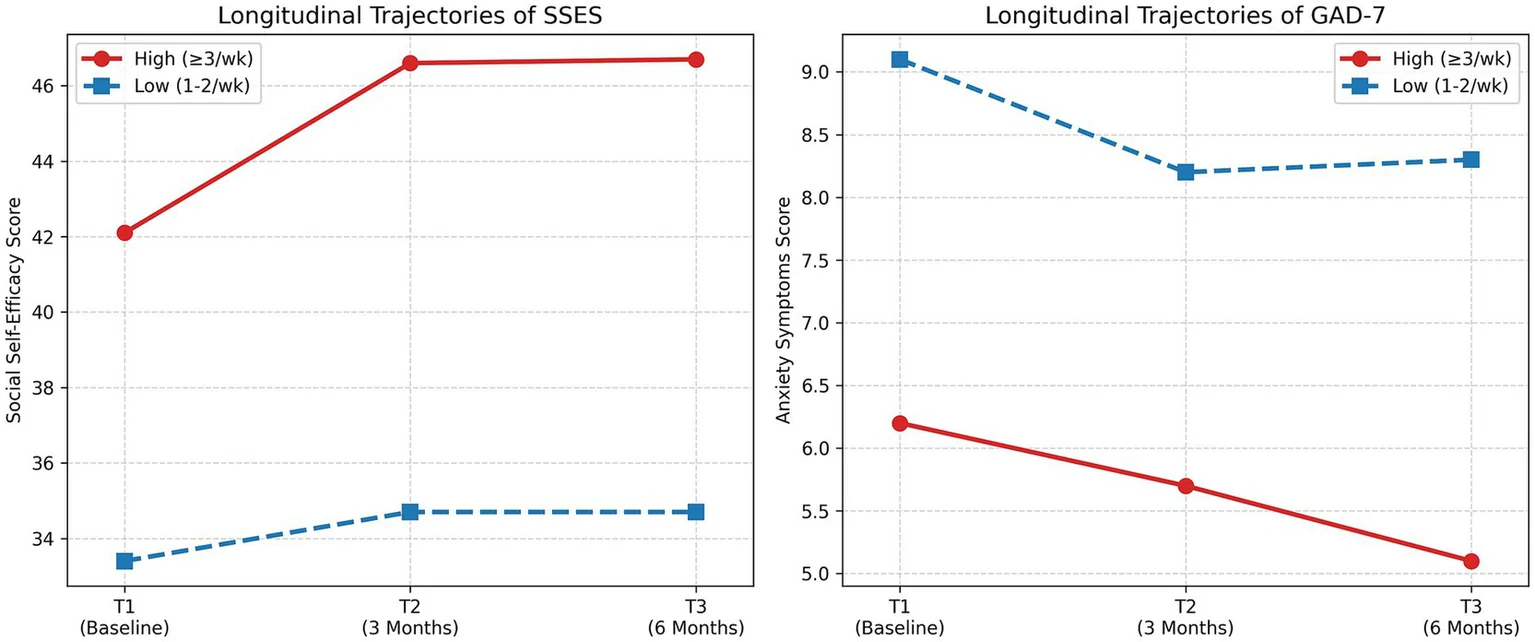
Longitudinal trajectories of SSES and GAD-7 by frequency group. The figure displays the three-wave trajectories of Social Self-Efficacy (SSES) and Anxiety Symptoms (GAD-7) separately for the high-frequency (≥3 sessions/week) and low-frequency (1–2 sessions/week) groups. Error bars represent 95% confidence intervals. The diverging patterns between groups over the six-month period illustrate the significant Time × Frequency Group interaction effects reported in the mixed-effects models. SSES showed progressive improvement in the high-frequency group with relative stability in the low-frequency group. GAD-7 showed gradual reduction in both groups, with a more pronounced decrease in the high-frequency group during the first 3 months.

Across all four outcomes, the high-frequency group consistently showed more favorable trajectories:

*GSES*: High-frequency group increased from 30.6 to 32.0 (+1.4). The low-frequency group remained stable (27.0 to 27.2, +0.2).

*SSES*: High-frequency group increased from 42.1 to 46.7 (+4.6). The low-frequency group showed modest increase (33.4 to 34.7, +1.3).

*GAD-7*: High-frequency group decreased from 6.2 to 5.1 (−1.1). The low-frequency group showed minimal change (9.1 to 8.3, −0.8).

*HRV*: High-frequency group increased from 49.4 ms to 54.3 ms (+4.9 ms). The low-frequency group remained stable (42.1 to 42.8, +0.7 ms).

Figure 3 presents the corresponding trajectories for GSES and HRV.

**Figure 3:**
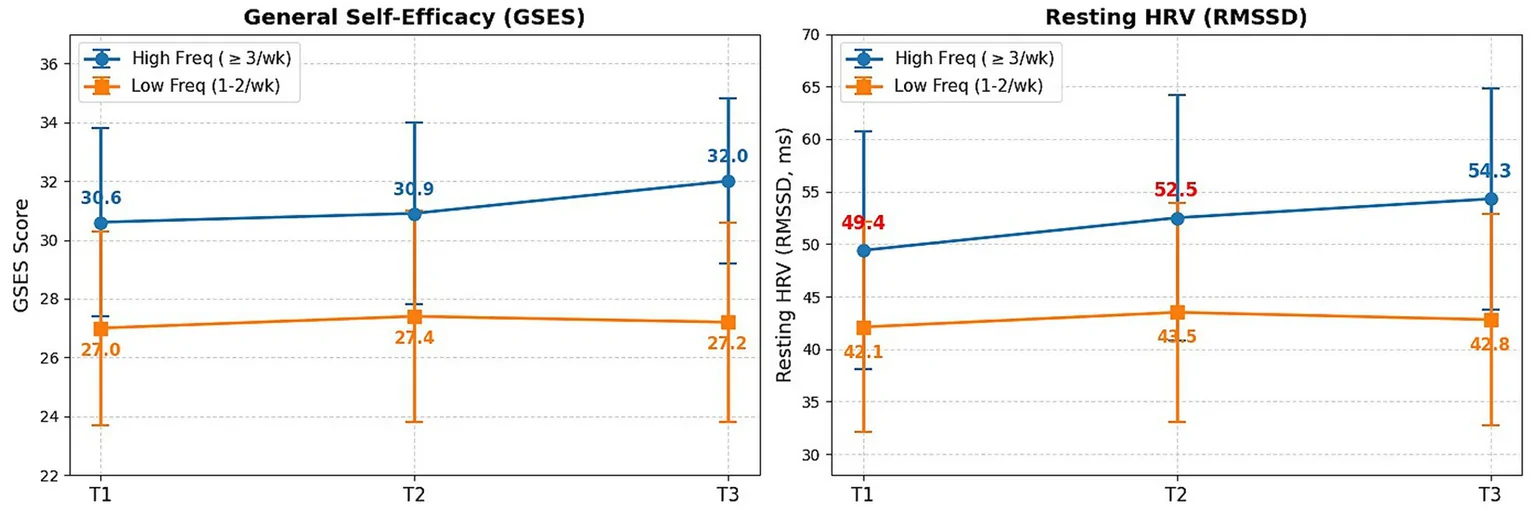
Longitudinal trajectories of general self-efficacy (GSES) and resting HRV (RMSSD) by frequency group. The figure displays the three-wave trajectories of General Self-Efficacy (GSES) and Heart Rate Variability (RMSSD) separately for the high-frequency (≥3 sessions/week) and low-frequency (1–2 sessions/week) groups. Error bars represent 95% confidence intervals. The patterns illustrate the significant Time × Frequency Group interaction effects reported for both outcomes at T3 (GSES: *β* = 0.99, *p* = 0.007; HRV: *β* = 3.10, *p* = 0.004), indicating that the high-frequency group continued to diverge from the low-frequency group over the six-month period.

### 4.4 Mixed-effects models

Linear mixed-effects models examined the effects of wave, baseline frequency group, and their interaction on each outcome, controlling for age, gender, and academic year. Table 5 presents the results of all four models in a consolidated format, with β coefficients, standard errors, and 95% confidence intervals for each predictor. Complete regression coefficients for the SSES model—including the intercept and all covariate effects—are provided in Supplementary Table S4.

**Table 5:** Results from linear mixed models examining the association between dance frequency group and longitudinal changes in outcomes (*N* = 152).

Predictor	GSES *β* (SE) [95% CI]	SSES *β* (SE) [95% CI]	GAD-7 *β* (SE) [95% CI]	HRV (ms) *β* (SE) [95% CI]
Wave T2 (vs T1)	0.43 (0.25) [−0.07, 0.93]	1.35 (0.51) [0.36, 2.35]**	−0.91 (0.21) [−1.33, −0.50]***	1.38 (0.75) [−0.09, 2.85]
Wave T3 (vs T1)	0.39 (0.28) [−0.16, 0.94]	2.47 (0.56) [1.36, 3.57]***	−1.35 (0.23) [−1.81, −0.89]***	1.38 (0.83) [−0.24, 3.01]
Freq_Group High (vs Low)	3.60 (0.54) [2.55, 4.65]***	8.62 (1.18) [6.30, 10.94]***	−2.80 (0.51) [−3.79, −1.81]***	7.64 (1.74) [4.23, 11.06]***
Wave T2 × Freq_High	−0.36 (0.35) [−1.05, 0.34]	2.40 (0.71) [1.00, 3.79]***	0.73 (0.30) [0.15, 1.31]*	1.29 (1.05) [−0.77, 3.35]
Wave T3 × Freq_High	0.99 (0.37) [0.28, 1.71]**	2.37 (0.73) [0.94, 3.81]**	0.48 (0.31) [−0.11, 1.08]	3.10 (1.08) [0.98, 5.22]**

*β* = unstandardized coefficient; SE, Standard error; values in brackets are 95% confidence intervals. All models adjusted for Age, Gender, and Academic Year (numeric). *p < 0.05, **p < 0.01, ***p < 0.001.

Random Effects: Intercept variance: GSES = 8.31, SSES = 42.37, GAD-7 = 7.82, HRV = 91.86. All models converged successfully.

Social Self-Efficacy (SSES). The model revealed a significant main effect of frequency group: high-frequency participants scored 8.62 points higher than low-frequency participants [*β* = 8.62, *p* < 0.001; 95% CI: (6.30, 10.94)]. Significant time effects indicated overall increases in SSES at T2 [*β* = 1.35, *p* = 0.008; 95% CI: (0.36, 2.35)] and T3 [*β* = 2.47, *p* < 0.001; 95% CI: (1.36, 3.57)]. Significant interaction effects emerged at T2 [*β* = 2.40, *p* = 0.001; 95% CI: (1.00, 3.79)] and T3 [*β* = 2.37, *p* = 0.001; 95% CI: (0.94, 3.81)], indicating that high-frequency participants showed greater SSES improvements over time.

General Self-Efficacy (GSES). The model showed a significant main effect of frequency group: high-frequency participants scored 3.60 points higher on GSES [*β* = 3.60, *p* < 0.001; 95% CI: (2.55, 4.65)]. No significant time main effects were observed. A significant interaction emerged at T3 [*β* = 0.99, *p* = 0.007; 95% CI: (0.28, 1.71)], indicating that high-frequency participants showed greater improvement in GSES by the six-month follow-up. The T2 interaction term was not significant [*β* = −0.36, *p* = 0.313; 95% CI: (−1.05, 0.34)].

Anxiety Symptoms (GAD-7). The model showed a significant main effect of frequency group: high-frequency participants scored 2.80 points lower on GAD-7 [*β* = −2.80, *p* < 0.001; 95% CI: (−3.79, −1.81)]. Significant time main effects indicated overall decreases in anxiety at T2 [*β* = −0.91, *p* < 0.001; 95% CI: (−1.33, −0.50)] and T3 [*β* = −1.35, *p* < 0.001; 95% CI: (−1.81, −0.89)]. A significant interaction emerged at T2 [*β* = 0.73, *p* = 0.014; 95% CI: (0.15, 1.31)], but not at T3 [*β* = 0.48, *p* = 0.113; 95% CI: (−0.11, 1.08)], suggesting that the anxiety-reducing effects of high-frequency participation may reach a plateau after 3 months. Age was also a significant predictor (*β* = −0.31, *p* = 0.038), with older participants reporting lower anxiety.

Resting HRV (RMSSD). The model showed a significant main effect of frequency group: high-frequency participants had 7.64 ms higher HRV [*β* = 7.64, *p* < 0.001; 95% CI: (4.23, 11.06)]. No significant time main effects were observed. A significant interaction emerged at T3 [*β* = 3.10, *p* = 0.004; 95% CI: (0.98, 5.22)], indicating that high-frequency participants showed greater improvement in HRV by the six-month follow-up. The T2 interaction term was not significant [*β* = 1.29, *p* = 0.220; 95% CI: (−0.77, 3.35)]. Gender was also a significant predictor (*β* = −3.84, *p* = 0.020), with males showing lower HRV than females.

Random Effects. Random intercept variances were as follows: GSES = 8.31, SSES = 42.37, GAD-7 = 7.82, HRV = 91.86. All models converged successfully.

### 4.5 Summary of quantitative findings

The longitudinal analyses revealed three key patterns.

First, a consistent baseline advantage for the high-frequency group across all four outcomes (all *p* < 0.001), with substantial effect sizes in SSES (*β* = 8.62), GSES (*β* = 3.60), HRV (*β* = 7.64 ms), and anxiety reduction (*β* = −2.80).

Second, significant Time × Frequency Group interaction effects indicated that high-frequency participants showed additional improvements over the six-month period beyond what would be expected from baseline differences alone. This pattern was most pronounced for SSES (significant at both T2 and T3), GSES (significant at T3), and HRV (significant at T3), while anxiety reductions appeared to plateau after T2.

Lastly, the dose–response pattern observed across all four outcomes supported a positive observational association between sustained dance participation and improvements in psychosocial and physiological wellbeing over time.

#### 4.5.1 Robustness check against selection bias

To evaluate whether the observed longitudinal benefits could be attributed to baseline differences rather than participation itself, we conducted propensity score matching and inverse probability weighting analyses. Prior to matching, standardized mean differences confirmed substantial baseline imbalance between frequency groups, most notably in SSES (SMD = 1.26), GSES (SMD = 1.11), GAD-7 (SMD = −0.96), and HRV (SMD = 0.68). After 1:1 nearest-neighbor matching, covariate balance was markedly improved, with all post-matching SMDs below 0.25 (Supplementary Table S5). In the matched sample, the advantage of the High-frequency group in SSES improvement (T3–T1) remained significant (*β* = 2.14, *p* = 0.006). This finding was corroborated by inverse probability weighting analysis on the full sample, which yielded consistent results. Taken together, these robustness checks indicate that the observed longitudinal benefits are not solely attributable to baseline differences.

#### 4.5.2 Sensitivity analysis for unmeasured confounding

To further assess the robustness of our findings to potential unmeasured confounding, we calculated E-values for the key estimates from the linear mixed models (31). The E-value represents the minimum strength of association (on the risk ratio scale) that an unmeasured confounder would need to have with both the exposure (dance frequency group) and the outcome to fully explain away the observed association.

For the main effect of High-frequency group on SSES, the E-value was 4.69, indicating that an unmeasured confounder would need to be associated with both Freq_Group and SSES with a risk ratio greater than 4.69 to explain away the observed difference. Similarly, the E-value for the Wave T3 × Freq_High interaction was 1.93. E-values for all key estimates are presented in Supplementary Table S6. These results suggest that our primary findings are unlikely to be fully explained by unmeasured confounding.

### 4.6 Qualitative findings

Four interconnected themes were developed from the interview and observational data. These themes provided process-oriented accounts that complemented the quantitative trajectories.

Theme 1: Rhythmic Entrainment as Affective Catalyst. Participants consistently described the Rock music as an active force shaping their engagement. The rhythm was described as something that “takes over” and provides relief from cognitive overcontrol. Participants noted that the immediate mood-elevating effect became more accessible over the six-month period.

“When the music starts, my body responds before I think. After a few months, I noticed I could reset much faster than before.” (P18, female, 21).

Theme 2: Embodied Mastery as Source of Self-Efficacy. The journey from awkwardness to fluid execution was described not as a single event but as a cumulative process. Participants distinguished this “body confidence” from academic achievement and noted that the mastery experience generalized to other domains over time.

“Learning a difficult sequence, failing, then finally getting it—that feeling stays with me. By the third month, I realized I had changed in ways I didn’t expect.” (P07, male, 22).

Theme 3: Synchronized Partnership as Context for Trust. Partner work was described as a “social training ground” where trust was built incrementally. The experience of moving in synchrony with a partner and with the group generated a sense of collective energy and belonging that deepened over time.

“At first it was awkward, but after dancing together for months, you learn to read each other. That trust builds slowly, then it becomes real.” (P11, female, 19).

Theme 4: Meaning-Making as Narrative Integration. Over the six-month period, participants integrated Dancesport into their personal identities. For some, it provided a stable community beyond their academic department. This ongoing narrative construction allowed participants to integrate disparate aspects of student experience into a more coherent self-narrative.

“Over six months, dancing to these songs has become a way to process things. It’s become part of who I am now, not just being a student.” (P25, male, 24).

## 5 Discussion

This three-wave longitudinal mixed-methods study examined whether Rock ‘n’ Roll Dancesport participation was prospectively associated with changes in self-efficacy, anxiety symptoms, and HRV among Chinese university students. The findings provide evidence that more frequent participation was associated with greater improvements across all four outcomes over 6 months. The Time × Frequency Group interactions were significant for SSES (at both T2 and T3), GSES (at T3), and HRV (at T3), indicating that high-frequency participants showed differential improvement beyond baseline advantages. The qualitative themes of rhythmic entrainment, embodied mastery, synchronized partnership, and meaning-making provided accounts of experiential processes through which participants perceived these changes to occur.

### 5.1 Interpretation of findings

Social Self-Efficacy. The association between high-frequency participation and greater improvements in SSES (*β* = 8.62 main effect; significant interactions at T2 and T3) is consistent with the inherently social nature of partner dance. The qualitative theme of synchronized partnership described how non-verbal communication, shared goals, and cooperative success build interpersonal trust and social confidence over repeated interactions. This finding extends the mediation model of Wang et al. (11) by suggesting that structured social practices may be particularly effective contexts for building relational resources through sustained engagement. The significant interaction effects indicate that this process continued to differentiate the two groups over time, rather than being fully accounted for by baseline differences.

Beyond individual confidence, what emerged from participants’ accounts was the reciprocal nature of the partner relationship—a process in which both individuals grow together, not in isolation but through mutual engagement. Partners offered each other support and encouragement through shared challenges and successes, creating a dynamic of co-development. This relational dynamic, characterized by trust-building and non-verbal attunement, may itself be a source of psychological resource accumulation, as partners not only learn to coordinate movements but also to sustain each other emotionally. Such interpersonal resource-building aligns with the mediation model of Wang et al. (11), extending it by suggesting that the social practice of partnered dance provides a context in which relational resources are co-constructed rather than merely individually acquired.

Anxiety Symptoms. The main effect of frequency group on GAD-7 scores (*β* = −2.80, *p* < 0.001) was substantial, representing a difference that is clinically meaningful for a self-report anxiety measure in a non-clinical population. The time main effects indicated overall reductions across both groups, but the significant interaction at T2 (followed by a non-significant interaction at T3) suggested that the anxiety-reducing effects of high-frequency participation may reach a plateau after 3 months. This pattern is consistent with the proposed affective mechanism of rhythmic entrainment: participants described the music as a reset from cognitive rumination, a process that may be most potent in the initial stages of engagement. This finding aligns with meta-analytic evidence that dance interventions significantly reduce depression symptoms, stress, and anxiety (8), with network meta-analyses ranking dance among the most effective non-pharmacological interventions for depressive symptoms (6).

General Self-Efficacy. The main effect of frequency group on GSES (*β* = 3.60, *p* < 0.001) showed that high-frequency participants entered the study with higher general self-efficacy. The significant interaction at T3 (*β* = 0.99, *p* = 0.007) suggests that the gap between groups widened over time, with high-frequency participants showing additional GSES gains by 6 months. This pattern is consistent with Bandura’s theory of self-efficacy (32), which posits that mastery experiences—such as progressively learning complex dance movements—are the most powerful source of self-efficacy beliefs. The qualitative accounts of embodied mastery as a cumulative process further support this interpretation: participants described body confidence that strengthened over time and generalized to other domains. Waugh and colleagues (33, 34) similarly found that dance self-efficacy influences both program participation and health outcomes, suggesting it may be a key mechanism driving health benefits. Koch, Morlinghaus, and Fuchs (35) demonstrated that a single dance intervention can significantly improve body self-efficacy and well-being, supporting the rapid affective benefits of dance engagement.

Heart Rate Variability. The main effect of frequency group on HRV (*β* = 7.64 ms, *p* < 0.001) showed that high-frequency participants had higher parasympathetic tone. The significant interaction at T3 (*β* = 3.10, *p* = 0.004) indicated further improvement over time. This finding is consistent with research by Zhao and colleagues (36), who demonstrated that dance rhythm significantly affects HRV during the recovery period. The 140 bpm rhythm condition in their study produced significantly lower HRV compared to 60 bpm and 100 bpm conditions (*p* < 0.05), suggesting that faster dance rhythms may place greater demands on autonomic regulation (36). Brown and colleagues (37) similarly found that HRV mobile technology can be used to individualize daily conditioning intensity for dancers, with the HRV-guided training group showing significant improvements in lower extremity power (*p* < 0.05) and trends toward greater muscle endurance over a 4-to-6-week period. These findings support the utility of HRV monitoring in dance populations for optimizing training adaptation and preventing overtraining (37). However, our interpretation requires caution: resting HRV increases with regular aerobic exercise generally, and this study did not assess participants’ overall physical activity outside of Dancesport. The HRV findings may therefore reflect general physical activity rather than dance-specific effects, as Laborde, Mosley, and Thayer (38) have noted that HRV is sensitive to numerous physiological and behavioral factors. Shaffer and Ginsberg (39) provided comprehensive normative data for HRV metrics, against which our sample’s values compare favorably.

### 5.2 Directionality and alternative explanations

A central concern in observational research concerns the direction of observed associations. The longitudinal design of this study provides some evidence for directionality, though it does not confirm causality. The Time × Frequency Group interactions indicate that high-frequency participants showed greater improvements over the subsequent three-to-six-month periods, suggesting that participation temporally preceded psychological gains. However, it remains plausible that participants with higher baseline self-efficacy and lower anxiety were more likely to maintain high dance frequency (self-selection), and that both processes operate simultaneously.

Three aspects of the data speak against a purely selection-based interpretation. First, the sample included 56 complete beginners (36.8%) and 63 participants with less than one year of experience (41.4%), indicating that Dancesport attracted individuals across a broad spectrum of baseline competence, weakening the plausibility of a pure self-selection account.

Second, the significant Time × Frequency Group interaction effects reported in Table 5 indicate that high-frequency participants gained additional improvements beyond their baseline advantages. If self-selection were the sole explanation, the two groups would be expected to maintain roughly parallel trajectories over time, with the baseline gap persisting but neither group gaining ground relative to the other. The observed divergence—particularly in SSES (T2: *β* = 2.40, *p* = 0.001; T3: *β* = 2.37, *p* = 0.001), GSES (T3: *β* = 0.99, *p* = 0.007), and HRV (T3: *β* = 3.10, *p* = 0.004)—is inconsistent with this expectation and instead suggests that participation itself contributed additional gains.

Lastly, the dose–response pattern across all four outcomes (Table 4) shows that higher participation frequency was associated with progressively larger improvements. In the high-frequency group, SSES increased by 4.6 points (vs. 1.3 in the low-frequency group), GSES by 1.4 points (vs. 0.2), and HRV by 4.9 ms (vs. 0.7 ms). This gradient is more consistent with a cumulative positive effect of participation than with a static difference determined at baseline. These quantitative patterns are corroborated by participants’ qualitative accounts, which described change and growth through the practice rather than merely affirming pre-existing traits, though these subjective reports should be interpreted with caution.

Nevertheless, several unmeasured factors may explain the observed associations. Individuals with more available time, higher socioeconomic status, or pre-existing social networks may both maintain higher dance frequency and show better psychological outcomes. The absence of a non-dancer comparison group further limits interpretation: we cannot distinguish whether the observed improvements are specific to Dancesport or would be observed with any regular structured activity. However, the E-value analyses suggested that unmeasured confounding would need to be implausibly strong (*E*-value = 4.47 for the SSES main effect) to fully account for these findings (Supplementary Table S6).

Furthermore, rhythmic competence alone does not predict sustained participation. Some participants in our sample exhibited strong rhythmic skills but low initial enthusiasm, while others had limited rhythmic coordination yet high emotional engagement—as captured in the qualitative theme of “rhythmic entrainment as affective catalyst.” This suggests that persistence is driven not by pre-existing musical aptitude, but by the affective resonance with the music, which deepens through repeated engagement Table 6.

**Table 6:** Qualitative themes and representative quotes.

Theme	Key Mechanism	Representative Quote
1. Rhythmic entrainment as affective catalyst	Physiological regulation; interruption of rumination	“When the music starts, my body responds before I think. After a few months, I noticed I could reset much faster than before.” (P18, female, 21)
2. Embodied mastery as source of self-efficacy	Somatic efficacy; cumulative confidence	“Learning a difficult sequence, failing, then finally getting it—that feeling stays with me. By the third month, I realized I had changed.” (P07, male, 22)
3. Synchronized partnership as context for trust	Non-verbal communication; shared agency	“At first it was awkward, but after dancing together for months, you learn to read each other. That trust builds slowly.” (P11, female, 19)
4. Meaning-making as narrative integration	Identity expression; emotional processing	“Over six months, dancing to these songs has become a way to process things. It’s become part of who I am now.” (P25, male, 24)

Quotations translated from Mandarin Chinese; minimal grammatical edits applied for clarity.

### 5.3 Cultural considerations

Dancesport originated in Western contexts, and we do not assert that it is inherently culturally consonant with Chinese values. However, its growing popularity among Chinese university students suggests that it has been adapted and adopted within local educational contexts. The qualitative themes of synchronized partnership and group belonging may resonate particularly strongly with collectivistic values, which emphasize interpersonal harmony, mutual reliance, and group cohesion. In the context of large, impersonal Chinese campuses, the intimate, non-verbal cooperation required in partner dance may offer a unique and potent counterbalance to social fragmentation, providing a “small community” experience that aligns with culturally valued ways of relating. However, this interpretation remains speculative and warrants direct empirical investigation.

Beyond cultural resonance, the connection between partners—encompassing both emotional attunement and physical expression—is itself a fundamental source of wellbeing. As participants in this study described, the gradual development of trust and non-verbal understanding through repeated partnered practice provided not only a sense of belonging but also a tangible experience of being seen, supported, and coordinated with another person. This relational-emotional-physical integration, which is central to partner dance, may be a key mechanism through which Dancesport contributes to psychosocial adaptation.

Future research should examine how cultural values shape the uptake and effects of arts-based physical activities in different contexts.

### 5.4 Strengths and limitations

Strengths of this study include the three-wave longitudinal design, which allowed examination of change over time; the mixed-methods approach, which provided both quantitative trajectories and in-depth experiential accounts; the inclusion of a physiological measure (HRV); and the focus on a culturally situated practice that is accessible and non-stigmatizing.

Limitations include the absence of a non-dancer comparison group; reliance on self-reported dance frequency and psychological measures; lack of control for overall physical activity outside of Dancesport; absence of direct measurement of positive affect, resilience, and interpersonal trust; the 5-min resting ECG recording, while fully adequate for time-domain RMSSD calculation per international guidelines (39, 40), is shorter than the 10-min recordings recommended for optimal frequency-domain analysis (HF, LF/HF). We therefore did not analyze frequency-domain HRV metrics and caution against overinterpreting spectral estimates from this protocol; and purposive sampling. Additionally, the GAD-7 measures anxiety symptoms, not clinical diagnosis; the term “anxiety” in this study refers to symptom severity. Attrition (21.1% over three waves, with 120 of 152 completing all three) is within acceptable range for longitudinal research, but may still introduce bias despite non-significant attrition analyses.

### 5.5 Practical implications

The findings suggest that Rock “n” Roll Dancesport is a promising candidate for further longitudinal and experimental evaluation. The dose–response pattern observed in both quantitative trajectories and qualitative accounts suggest that sustained engagement, rather than occasional participation, is associated with greater benefits. These findings align with systematic review evidence that dance interventions can have positive effects on aspects of the participants’ self, including self-concept and self-esteem (22). Institutional support for student-led arts-physical communities may be warranted, provided that participation is voluntary and the focus remains on enjoyment and community rather than performance pressure. Causal confirmation requires randomized controlled trials or quasi-experimental designs with non-dancer control groups.

## 6 Conclusion

This three-wave longitudinal mixed-methods study found that more frequent Rock “n” Roll Dancesport participation was prospectively associated with improvements in social self-efficacy, general self-efficacy, resting HRV, and reductions in anxiety symptoms among Chinese university students over 6 months. The Time × Frequency Group interactions provided evidence that high-frequency participants showed greater improvements over time, suggesting that participation temporally preceded psychological gains. Qualitative accounts of rhythmic entrainment, embodied mastery, synchronized partnership, and meaning-making provided process-oriented explanations consistent with the quantitative trajectories. These findings highlight the potential value of structured, arts-based physical activities as a non-stigmatizing context for resource-building among university students, while underscoring the need for controlled designs to establish causality. Understanding the organic, culturally situated practices through which students navigate stress may inform the development of proactive wellbeing strategies that complement traditional clinical services.

## Data Availability

The original contributions presented in the study are included in the article/Supplementary material, further inquiries can be directed to the corresponding author.
