# Supplementary material for: Dancing to wellbeing: a three-wave longitudinal mixed-methods study of Rock “n” Roll Dancesport and psychosocial outcomes in Chinese university students
Source: Front Public Health. 2026 Oct 2;14:1928623. doi: 10.3389/fpubh.2026.1928623 (PMC13674341; doi:10.3389/fpubh.2026.1928623)
Supplement: Supplementary file 1 [file Table_1.DOCX]

**Supplementary Tables (S1–S6)**

**Baseline Comparisons Between High- and Low-Frequency Groups**

| Variable | High (n = 84) | Low (n = 68) | p (t-test) |
| --- | --- | --- | --- |
| GSES | 30.6 ± 3.2 | 27.0 ± 3.3 | < .001 |
| SSES | 42.1 ± 6.2 | 33.4 ± 7.6 | < .001 |
| GAD-7 | 6.2 ± 2.8 | 9.1 ± 3.4 | < .001 |
| HRV | 49.4 ± 11.3 | 42.1 ± 10.0 | < .001 |

*Note.* Values are presented as Mean ± SD. High = ≥3 structured sessions per week; Low = 1–2 sessions per week. GSES = General Self-Efficacy Scale; SSES = Social Self-Efficacy Scale; GAD-7 = Generalized Anxiety Disorder 7-item scale; HRV = Heart Rate Variability (RMSSD).

**Table S2**

**Baseline Correlations Among Key Variables at T1 (Pearson r)**

| Variable | GSES | SSES | GAD-7 | HRV |
| --- | --- | --- | --- | --- |
| GSES | 1.00 | 0.34*** | −0.18* | 0.20* |
| SSES | 0.34*** | 1.00 | −0.28*** | 0.09 |
| GAD-7 | −0.18* | −0.28*** | 1.00 | −0.29*** |
| HRV | 0.20* | 0.09 | −0.29*** | 1.00 |

*Note.* *p < .05, ***p < .001. SSES–HRV correlation is non-significant (p = .286).

**Table S3**

**Normality Tests (Skewness and Kurtosis) for All Outcome Variables Across Waves**

| Variable | T1 Skew / Kurt | T2 Skew / Kurt | T3 Skew / Kurt |
| --- | --- | --- | --- |
| GSES | −0.10 / −0.40 | 0.08 / −0.17 | −0.37 / −0.48 |
| SSES | −0.25 / −0.19 | −0.14 / −0.22 | −0.40 / −0.10 |
| GAD-7 | 0.16 / 0.38 | 0.04 / −0.40 | 0.29 / −0.22 |
| HRV | 0.09 / −0.07 | 0.10 / −0.11 | 0.08 / −0.14 |

*Note.* Kurtosis values are excess kurtosis (Fisher's definition). All |skew| and |kurtosis| < 1, supporting approximate normality for LMM assumptions.

**Table S4**

**Complete Regression Coefficients for Social Self-Efficacy (SSES) Model**

| Predictor | β | SE | z | p | 95% CI |
| --- | --- | --- | --- | --- | --- |
| Intercept | 25.04 | 7.43 | 3.37 | .001 | [10.47, 39.61] |
| Wave T2 | 1.35 | 0.51 | 2.67 | .008 | [0.36, 2.35] |
| Wave T3 | 2.47 | 0.56 | 4.39 | < .001 | [1.36, 3.57] |
| Freq_High | 8.62 | 1.18 | 7.29 | < .001 | [6.30, 10.94] |
| Wave T2 × Freq_High | 2.40 | 0.71 | 3.37 | .001 | [1.00, 3.79] |
| Wave T3 × Freq_High | 2.38 | 0.73 | 3.24 | .001 | [0.94, 3.81] |
| Age | 0.31 | 0.35 | 0.89 | .374 | [−0.38, 1.00] |
| Gender_Male | −0.55 | 1.12 | −0.49 | .621 | [−2.74, 1.64] |
| Academic_Year_Num | 0.95 | 0.55 | 1.72 | .086 | [−0.13, 2.03] |

*Note.* Estimated via the MIXED procedure in SPSS (version 28.0) using REML estimation. Random intercept variance = 42.37.

**Table S5**

**Covariate Balance Before and After Propensity Score Matching**

| Covariate | Pre-matching SMD | Post-matching SMD |
| --- | --- | --- |
| SSES_T1 | 1.26 | ~0.08–0.18 |
| GSES_T1 | 1.11 | ~0.08–0.19 |
| GAD-7_T1 | −0.96 | ~−0.22–0.21 |
| HRV_T1 | 0.68 | ~−0.06–0.15 |
| Age | 0.34 | ~−0.21–0.11 |
| Dance Tenure | 0.14 | ~−0.12–0.07 |

*Note.* SMD = standardized mean difference (pooled SD). Post-matching example uses 1:1 nearest-neighbor matching on logistic propensity score with caliper ≈ 0.2 SD of the logit-transformed propensity score. Exact post-matching values depend on the matching algorithm details.

**Table S6**

**E-values for Key Estimates from Linear Mixed Models**

| Effect (from Table 5) | β (SE) | Approx. d | Approx. RR | E-value | Interpretation |
| --- | --- | --- | --- | --- | --- |
| SSES Freq_High main effect | 8.62 (1.18) | 1.06 | 2.63 | 4.69 | Very robust |
| SSES Wave T3 × Freq_High | 2.37 (0.73) | 0.29 | 1.30 | 1.93 | Moderately robust |
| GSES Freq_High main effect | 3.60 (0.54) | 0.97 | 2.41 | 4.26 | Very robust |
| GAD-7 Freq_High main (protective) | −2.80 (0.51) | 0.83 | 2.12 | 3.66 | Robust |
| HRV Freq_High main effect | 7.64 (1.74) | 0.67 | 1.85 | 3.09 | Robust |

*Note.* E-values calculated using the method of VanderWeele and Ding (2017). Cohen's d approximated as |β| / SD_T1 of the outcome. RR ≈ exp(0.91 × d). Higher E-values indicate greater robustness to unmeasured confounding.
